# A policy review on the visibility of migrant women exposed to, and at risk of gender-based violence: Considerations for inclusive and equitable policies and programs in Canada

**DOI:** 10.1371/journal.pgph.0002919

**Published:** 2024-02-16

**Authors:** Cyndirela Chadambuka, Beverley Essue

**Affiliations:** Institute of Health Policy Management and Evaluation, Dalla Lana School of Public Health University of Toronto, Toronto, Canada; University of Washington Department of Global Health, UNITED STATES

## Abstract

Gender based violence (GBV) has had distinct and disproportionate impact on the health and wellbeing of migrant women in Canada. Currently, there is dearth of documented information concerning the inclusion of migrant women in GBV-related public policies in Canada. The present study examines the extent to which Canadian public policies reflect and address the needs of migrant women who have experienced GBV. We conducted a policy review of publicly available documents at federal and provincial (British Columbia and Ontario) levels. Migrant women’s visibility in the Canadian policy landscape remains limited, as their unique needs are often grouped into broader categories such as ‘newcomers, or visible minorities’. This approach fails to acknowledge their distinct lived experiences. Regardless of the federal and provincial efforts in developing policies targeted at GBV prevention, several significant policy gaps came to light. These include the absence of well-defined protective measures for migrant women in precarious employment as well as the hurdles posed by immigration restrictions that pose a significant challenge for those seeking to leave abusive relationships, particularly in cases of dependent migration status. The highlighted policy gaps have negative impact on migrant women’s social functioning, including limiting access to services and opportunities, and this has adverse effects on their overall health and wellbeing. To ensure the effectiveness and significance of GBV policies, it is imperative that policymakers acknowledge and target the distinct vulnerabilities and needs of migrant women who experience GBV. The inclusion of an intersectional perspective in the formulation and implementation of policies is essential, as it facilitates the recognition of the distinct needs of migrant women. Failure to acknowledge these varying needs and the real-life experiences of this diverse group can jeopardize the comprehensive and successful implementation of GBV response policies, not only in Canada but also worldwide. Prioritizing this aspect is crucial.

## Introduction

Gender-based violence (GBV) is a significant threat to the wellbeing of women, a violation of human rights and a widespread public health crisis in Canada. Previous research conducted has revealed that one in three women experience GBV in her lifetime [1, 2]. GBV is defined as any harmful act that is perpetrated against a person based on their gender in public or private spaces and includes inflicting physical, sexual, and mental harm or suffering and deprivation of liberties [3]. Importantly, GBV includes partner (commonly referred to as intimate partner violence (IPV) or domestic violence) and non-partner violence.

In Canada, the prevalence of GBV remains high with four in 10 women reported to have experienced it, with 44% of women experiencing IPV and 30% sexual assault since the age of 15 [4, 5]. Canada has supported international GBV prevention initiatives and signed conventions aimed at ending discrimination and violence against women. For example, Canada ratified the Convention on the Elimination of All Forms of Discrimination against Women (CEDAW) (1979) and put in place laws and policies that criminalize any behavior aimed at threatening or harming another individual. While this broader governance framework is crucial in the prevention of GBV, the Canadian policy landscape reflects barriers to policy implementation that can limit the reach and effectiveness of these conventions for all individuals affected by GBV [6]. Ultimately the risk of exposure to, and tragic consequences of, GBV still persist in Canada and many subgroups of women face disproportionately higher risks of violence.

Canada stands out as welcoming to immigrants from all over the world [7]. Immigrants are considered a major source of trained labor that is believed to sustain Canada’s economic growth and labor shortages and ultimately contribute to achieving the United Nations Sustainable Development Goals (SDGs) [8, 9]. It is reported that by 2036, immigrants could constitute 30% of the Canadian total population, a steep rise in immigration [5]. The Canadian immigration system categorizes migrants into three broad classes namely; economic class, which also includes temporary workers and students; family members; and refugees [8, 10]. In most cases, men play a predominant role as the primary actors in migration. In the context of heterosexual relationships, when men are viewed as the main actors in migration, this can put women in a precarious position and increase their susceptibility to abuse [11].

Upon migrating to Canada, many migrants, particularly migrant women, find themselves starting their careers and lives from scratch. A study by Momani and colleagues [12] revealed that migrant women face challenges in the labour market regardless of their education and skills acquired in their home countries. The mammoth task of translating their skills and education to the Canadian labour markets is daunting and demotivating. Similarly, Warman and colleagues [13] argue that the match between migrant women’s pre-immigration occupations with similar occupations in Canada potentially increases their productivity, thereby influencing both health and non-health outcomes. However, having access to skilled and secure employment does not make migrant women less vulnerable to GBV, given the myriad of intersecting socio-economic, and cultural factors that increase their vulnerability [14].

While it is well understood that people migrate to overcome poverty, conflict or cope with economic and social instability, the gender norms that migrants may subscribe to in their home countries can create vulnerabilities and inequalities that provide a ‘breeding’ ground for GBV perpetration [9]. Many migrant women were born in countries with histories marked with political violence, and cultural norms that prioritizes men’s rights and condone the subjugation of women. As argued by Corley and Sabri [15], research usually focuses on migration as the starting point of analysis on GBV, without considering the pre-migration and post-migration experience of women. Pre-migration stressful experiences can intersect with post-migration experiences, impacting the overall health and wellbeing of women [16]. Understanding the pre-migration and post-migration experiences is crucial as in some instances GBV can be either a pull factor or a consequence of migrating. For example, in a study with African immigrants in the United States (US), conducted by Corley and Sabri [15], IPV was reported as a push factor to migrating to the US, while for others it was a consequence of the increased vulnerability following migration on a spousal visa.

The advent of COVID-19 has been regarded as a vector in deepening the social and economic inequalities that exacerbate the risk for migrant women to GBV [17]. COVID-19 interrupted the social system through the introduction of lockdown measures which led to isolation and limited access to care and support especially for migrant women -all linked to the increase in GBV cases [18]. Consequently, the need for IPV services has increased since the COVID-19 pandemic and this signifies the importance of promoting increased support for GBV intervention initiatives that help to address the many (unique) competing needs of migrant women in the wake of any pandemic.

The gendered nature of migration coupled with the marginalization and discrimination of poor racialized migrant women further increase their risk to abuse and reduces their ability to seek support [19–21]. This underscores how the intersection of multiple systems of oppression and inequality place migrant women at a higher risk of abuse as compared to non-racialized migrant women. The theory of intersectionality argues that people who are marginalized in multiple ways (e.g., racialized migrant women) are likely to experience oppression differently as compared to women marginalized in one dimension [22]. Intersectionality further cautions us against using single factor explanations to understand individual experiences of violence but rather seek to understand women through their individual or group experiences [23, 24]. There is a need to recognize the heterogeneity of migrant women’s circumstances and experiences of GBV which is dictated by both their pre-migration and post-migration contexts.

Considerable attention has been drawn to the scope and extent of IPV against migrant women in Canada [8, 25, 26] yet the breadth of GBV exposures and the negative consequences extend beyond partner violence. There is a dearth of literature on the extent to which GBV- related policies address the needs of migrant women. Studies that holistically focus on the abuse of migrant women (including non-partner violence) are crucial for crafting interventions that are specific to the needs of this marginalized population in Canada, with relevance to other contexts with large migrant populations. Thus, this policy review sought to fill this gap. Examining the current Canadian policy landscape provides us with an opportunity to understand the tensions and gaps that exist within GBV-related policies not only in Canada but globally. This review foregrounds the significance of context-specific policies that respond to the unique needs of migrant women exposed and at risk of GBV and the goal of contributing to promoting their wellbeing. In this policy review, the focus was not only on policies that are specific to GBV but also included policies that focus on access to determinants of safety of GBV survivors (e.g., housing and labor policies). Two research questions guided this policy review.

1. How is GBV reflected in policies that relate to violence against women?
2. How are migrant women accounted for in policies that relate to GBV in Canada- at federal and provincial level (British Columbia and Ontario)?

## Methods

This policy review focused on federal and a subset of subnational policies on violence against women. Two provinces were selected: British Columbia (BC) and Ontario (ON), because they attract the highest proportion of immigrants (40.8% and 46.1%) in Canada [5]. In Canada, the federal government has jurisdiction and can enact policies and legislation to the entire country. Each provincial government has jurisdiction over its provincial boundaries and population. Provincial governments are not entirely subordinate to the federal government and have areas of jurisdiction specifically assigned to them.

### Policy frameworks

This policy review was guided by the Intersectional Feminist Policy Analysis (IFPA) Framework [22] and the Health Policy Triangle (HPT) Framework by Walt and Gilson [27].

#### The Intersectional Feminist Policy Analysis (IFPA) framework

This framework comprises various questions that are intended to interrogate policy during the review process. The questions are grouped according to major themes of feminist analysis and include *equality; gender neutrality; multiple identities; context of the policy*. Although this framework is mainly pursued to bring equality between men and women it also seeks to bring a balance between privileged and oppressed women [22, 28]. In this framework ‘oppressed’ is understood as a systematic denial which negatively impacts people’s ability to access resources, services of their surrounding communities for their wellbeing [22]. As such, it gives us an opportunity to understand migrant women’s vulnerabilities, the different forms of varying precarity around immigration status, stereotypes, prejudices about migrant women and the different ways inequality can be perpetuated or mitigated in policy formulation and implementation. In this study, the IFPA framework allows us to think about the extent to which policies have responded to the issue of GBV against migrant women, how their needs are addressed in the policies, and how policies maintain or create inequities between different groups of women.

#### Health policy triangle framework

The HPT framework systematically reveals complex interrelationships in policy making and implementation [29]. The HPT framework focuses on *actors* (influential individuals and organizations); the *context* (political, economic etc.); *the content* (policy objectives, guidelines) and the *process* (ways in which policies are developed) (see Table 1)). [29] further argue that health policy analysis largely focuses on the content and neglects actors, context, and process. Thus, the HPT framework is useful as it will allow us to analyze the interconnectedness of the different categories and how they influence the extent to which these policies respond to the needs of migrant women.

**Table 1:** Policy frameworks.

IFPA Framework themes	HPT framework elements
Intersectional Identities	Actors
State market Control	Context
Equality	Process
Gender Neutrality	Content
Context and visibility	
Equity and care	

### Inclusion and exclusion criteria

We reviewed policies that are publicly available on the federal and provincial websites. The inclusion criteria for the publicly available policy documents (federal and provincial) included:

- Target: individuals who identify themselves as women (transwomen and cisgender included) should be the target of the policy or should be included in the target population
- Focused on prevention and response to violence against women (including GBV, intimate partner violence, sexual violence, technology facilitated violence, human trafficking) against women in Canada.
- Policies not specifically on violence against women but whose objectives and aims promote (direct or indirectly) prevention and response to GBV against migrant women (e.g., health policies, immigration policies, labor policies, housing policies)

### Policy searches and search terms

We conducted a policy search on Google, federal, and provincial websites. On the federal website, we searched for selected policies in each relevant government agency website. Key words such as ‘violence’ or ‘GBV’, ‘intimate partner violence, ’family violence’, ‘domestic violence’, ‘spousal abuse’, ‘migrant’, ‘immigrant’, ‘minority groups’ and ‘ethnic minority groups’ were used to conduct the policy search at federal level. These key words were then adapted for conducting policy search for BC and ON.

### Data analysis

Data was extracted at federal and provincial level. The HPT framework was used for data collection and extraction. We utilized the elements of the HPT framework to classify extracted data and to analyze the actors involved in the consultation process (where applicable) and the stated process of policy implementation. This process was done for each policy at both federal and provincial levels. Thereafter, the IFPA framework was utilized to critique the policies, to determine the extent to which they reflect and address the needs of migrant women. We first conducted the analysis at the federal level and then we examined policies for each province using the themes and questions in IFPA framework to understand the different gender dimensions and the inclusion of the needs of migrant women in the policy. As the IFPA framework uses a holistic lens in analyzing the interconnectedness of different components of lived experiences of women, not all themes/questions from the framework were used to critique the policies but only those key themes that align with the objectives of this policy review (see Table 1).

## Results

A total of 37 GBV-related policy documents were identified and reviewed (S1 Table). At the federal level, 14 policies were identified, and 12 for British Columbia (BC) and 11 for Ontario (ON). All the policies reviewed in this paper were current, as of the search date.

### Conceptualization of GBV in the policies reviewed

To better understand how GBV was accounted for in the policies, we focused on the terms that were used to refer to GBV. We noted that the terms used in the Canadian policy landscape are similar to terms (i.e., GBV and violence against women) used by the international community to capture the nature of violence as outlined in the United Nations Declaration on the Elimination of Violence against Women (1993). At both federal and provincial levels, public policies related to GBV use various terms such as ‘domestic violence’, IPV, ‘GBV’ and ‘family violence’ to refer to abusive behaviors against individuals perpetuated within the context of an intimate relationship with the perpetrator. The use of these terms also reflects the different ways GBV manifests itself. Domestic violence (or IPV) and sexual assault are the most addressed forms of GBV in both federal and the selected provincial public policies. Notably, trafficking, and cyber-violence or technology-facilitated violence are recognized as criminal offenses and forms of abuse at federal levels through the Criminal Code and Protecting Canadians from Online Crime Act yet in provincial policies there is limited information on them.

### Federal and provincial policies through an intersectional lens

We analyzed the policy documents in relation to the HPT and IFPA Frameworks and our results were categorized according to the six themes of the IFPA framework.

#### Intersectional identities

The concept of intersectional identities received considerable attention in the federal policies that were reviewed. There was emphasis and acknowledgement of the different social classes that women occupy and the multiple identity markers that shape the lived experiences of migrant women exposed and at risk of GBV. Federal policies such as Its Time: Canada’s Strategy to prevent and address GBV (hereinafter referred to as The Strategy), and A Place to Call Home: National Housing Strategy, emphasize the need to prioritize the needs of migrant women and ensure that their needs are addressed in the provision of support and services regardless of gender, sex, and nationality. The contribution of pre-migration and post-migration conditions that intersect in exacerbating migrant women’s risk to GBV are also articulated in The Bill S7 Zero Tolerance for Barbaric cultural practices where the federal government commits to protect migrant women from harmful practices that threaten their health and wellbeing.

The subsequent development of provincial GBV-related policies also took into consideration the multiple identities of women impacted by GBV and their needs regardless of their racial background, gender, and sexual orientation. Many policies acknowledge the intersecting identity markers and ensure service provision is extended to migrant women experiencing GBV and resident in the two provinces. For example, the Ontario Works Act, Housing Services Act (2011), Victims Bill of Rights (1995), BC Housing and Women’s Transition housing and Support Program, and Victim Link BC ensure services are available to vulnerable women and denounce discrimination based on gender, immigration status or race. BC also put measures in place, through the Crime Victim Assistance Program, to ensure that all GBV survivors get adequate support regardless of gender, sexuality, race, or immigration status. Information regarding the accessibility of support and care is available in several languages, for example, Filipino, Punjabi, and Chinese. Interestingly, there appears to be an implicit assumption that migrant women’s experiences are the same with other Canadian-born racialized populations as their needs are bracketed with those of other minority populations.

#### State-market control

Most federal policies reviewed touched on the aspect of promoting economic participation of migrant women exposed and at risk of GBV. Measures have been put in place to ensure that migrant women exposed to GBV are still able to participate in economic activities by prioritizing the health and safety of employees regardless of gender, racial identity, or immigration status. To illustrate, through the Canada Labor Code and the Workplace Harassment and Violence Prevention Regulations of 2020, the federal government has put forward measures to address employment standards, protect all employees from workplace sexual harassment and promote economic participation. Employers are required to prioritize prevention and response to workplace violence by having a workplace harassment and violence prevention policy articulating the procedures to be followed in the event of harassment and violence. There is a consistent emphasis on the need to have policy measures in workplaces that ensure that victims of domestic violence get access to support and care while maintaining their employment or source of income.

At the provincial level, the government of ON and BC have followed suit and put in place policies and legislative measures with the core aim of protecting the rights of employers and employees in workplaces and ensuring that domestic violence victims are protected from abuse and violence that may lead to physical injuries and have access to care and support while maintaining their employment. For example, the Employment Standards Act, and Occupational Health and Safety Amendment Act (Violence and Harassment in Workplace) make provisions of protected leave for victims of domestic violence by providing 10 days to 15 weeks in one calendar year to facilitate access to care and support for the victims and their children. In BC, victims of domestic and sexual violence have access to protected leave through the Domestic and Sexual Violence Leave which stipulates that “an employee can take up to 5 days of paid leave and 5 more days of unpaid leave per calendar year if they are impacted by domestic or sexual violence. If necessary, an employee can take up to 15 more weeks of unpaid leave”. Importantly, there are no clear provisions on some industries offering precarious employment (e.g., personal support workers, hospitality, and tourism industry) and impacted by COVID-19 on the extent to which they are covered by the policies. This lack of clarity may mean that policy provisions might end up not benefitting most migrant women in precarious employment and at risk of and exposed to GBV.

#### Equality

Equality goes beyond dismantling gender inequality but also includes oppressions rooted in the intersection of multiple disadvantages such as race, class, and immigration status which can compound gender disadvantages. Analysis of the policies reviewed at the federal level revealed Canada’s commitment to promote equality in the prevention of GBV. To illustrate, The Strategy strengthened the federal government’s commitment to promote equality and the eradication of GBV. Furthermore, through the Canadian Human Rights Act, The Labor Code, and the Poverty Reduction Strategy, the federal government puts in place initiatives to eradicate inequalities based not only on gender but on multiple identities that increase vulnerable women’s risk to violence. Noteworthy in these policies is the clear emphasis on the need to eliminate discrimination against individuals from minority groups (migrant women included) based on their color, ethnicity, sexual orientation, immigration status, religions, or national origin.

At the provincial level, there is evidence of promotion of equality in the current policies. For example, the Employment Standards Act, Employment Protection for Foreign National Act and Housing Services Act in ON, ensures that the needs of women from vulnerable populations are prioritized and strives to protect their interests. Likewise, BC has made progress to ensure that gender differences and the resultant vulnerabilities that place women from minority groups at a higher risk of GBV are reflected in the provincial public policies. For example, the Domestic and Sexual Violence Leave has been put in place to ensure that women who are exposed and at risk of violence are not deterred from labour participation, and are allowed to seek care and support, even for their children affected by the abuse.

#### Context and visibility of women

In selected policies, context, and visibility of migrant women refers to acknowledgement of the economic and social realities (in the countries of origin and destination) that shape their lived experiences of GBV (whether location, culture, or institution specific). Through the Bill S7 Zero Tolerance for Barbaric Cultural Practices Act (2015), the federal government demonstrates its commitment to eradicate all forms of violence against women in the name of cultural practices and honor (e.g. female genital mutilation, honor killings)- practices that are common in most migrant women’s country of origin. Furthermore, some federal legislations were amended to demonstrate Canada’s intolerance of the such harmful practices; i) Immigration and Refugee Protection Act- now regard polygamy as grounds for inadmissibility and removal for permanent and temporary residents (ii) Civil Marriage Act-now promotes free and enlightened consent and (iii) Criminal Code- is committed to the prevention of forced marriages common among migrant women under dependency status.

While there are no specific policies focusing on the cultural realities shaping GBV experiences in BC and ON, we identified policies that are built on the federal government’s policies committed to the prevention and response to GBV through providing access to care and support. For example, the Victim Bill of Rights (ON), Occupational Health, and Safety Amendment Act (Violence and Harassment in Workplace) (ON) are designed to protect migrant women exposed and at risk of GBV in workplaces. Additionally, the BC Housing—Women’s Transition Housing and Support Program, is dedicated to providing safe housing for GBV victims and their children.

Further, the needs of women from minority groups are visible as their insights are incorporated through a consultative process in developing various initiatives. The Domestic and Sexual Violence Leave in BC and initiatives such as VictimLink BC also provide information, referral services and immediate crisis support in 150 languages. ON policies such as The Poverty Reduction Act clearly states that poverty reduction strategies, “must recognize the heightened risk among groups such as immigrants, women, single mothers, people with disabilities, aboriginal peoples, and racialized groups”. This clearly denotes the inclusion of minority women including migrants who are at a heightened risk of GBV.

#### Equity and responsibility

The principle of equity promotes fair conditions for all people to fully participate in society. The adoption of a gender neutrality stance in the federal policies dismantled the pattern of men being viewed as the public actors while women remain passive actors whose main role is caregiving. When women assume the role of passive actors, it makes them invisible, their needs not recognized and ultimately their abuse is shrouded in the culture of silence. The federal government has recognized the multiple roles that women have (caregiving roles versus labor participation and the associated abuse) and has made strides to develop policies that ensure women are able to participate in the workforce even when they are exposed and at risk of GBV. Although the federal childcare initiative, Toward $10-a-Day: Early Learning and Childcare, is still in its early stages and yet to be fully implemented, its aim is to make childcare more affordable thereby making it possible for migrant women exposed to GBV to seek care and support and not be deterred by childcare duties or work responsibilities.

The adoption of the Toward $10-a-Day: Early Learning and Child Care in ON and BC also eases the burden of caregiving, providing migrant women exposed and at risk of GBV the opportunity to access care and support when needed. Additionally, the BC government has the Affordable Child Care Benefit initiative which is a monthly payment to help eligible family members with the cost of childcare. Eligibility is dependent on income, family size and the type of care and residency status (Canadian citizens, permanent residents, convention refugees and persons in need of protection are eligible). However, it is not clear which ‘person in need’ is eligible and the policy excludes temporary residents on work and study permits who are equally vulnerable to GBV. Although the ON Health Insurance Program and the BC Government Medical Services Plan (BC MSP) aims to promote access to healthcare, we identified inadequacies (long waiting lists and eligibility factors linked to supporting documentation) that may compromise access to health services for migrant women exposed to GBV who might need urgent access to healthcare and non-healthcare services.

#### Gender neutrality

Gender neutrality has been at the core of policy reforms at the federal level, and synonymous with the goal of equality in the prevention of GBV. The federal government has ensured that policies that are developed are gender neutral and do not discriminate against victims of GBV. This is illustrated through reforms in the language used in the policies that denote the visibility of migrant women and their experiences of GBV. The federal government has advocated for the use of languages that are not gender specific with the aim of reducing barriers to equal access to care and support for victims of GBV. For example, the use of “persons” instead of “men and women” in the Canada Labor Code; “foreign national” in the Immigration and Refugee Protection Act to refer to both male and female migrants.

At the provincial level, the Ontario Human Rights Code also denounces discrimination and sexual harassment based on gender, sexual orientation, and nationality among others and the use of gender-neutral languages is visible in most policies to ensure that all “persons” exposed to GBV can equally access support services as required regardless of nationality or sexual orientation. Furthermore, the Ontario Police Services denounce racial and gender profiling of perpetrators with the aim of protecting mainly victims from racialized populations. BC also adopted gender neutrality in the GBV related policies by ensuring that all “persons’ vulnerable to GBV have access to support services that are required and removed approximately 750 gendered languages in 70 BC laws and regulations to accommodate all vulnerable persons in their policy framework.

### Key policy gaps identified

Based on our analysis, we identified key policy gaps that may negatively impact the fabric of support for migrant GBV survivors in Canada and ultimately the wellbeing of migrant women.

#### Categorization of migrant populations as racialized/minority groups in policies

In most policies at the federal and provincial levels, migrant women are ‘bundled’ with racialized women and their needs are equated with those of non-racialized women regardless of their unique lived experiences of GBV. As argued by researchers [30], the migrant population is largely indistinguishable from Canadian-born racialized/minority groups in most policies. There is mostly racial or ethnic, or gendered categorization of women in policies, which does not reflect some aspects of migrant women (e.g., cultural aspects, nationality, or first generation or second-generation immigrants, length of stay in Canada). For example, newly-arrived migrant women exposed to GBV in Canada may have different needs as compared to those who have stayed longer and some benefits and social rights for migrant survivors vary according to legal status and length of residency in Canada [31]. Blanketing the needs of migrant women, who in most cases are multiply minoritized based on racial and ethnic background, gender, immigration status, education level, under the racialized/minority category promotes a ‘one size fits all’ intervention approach that might fail to capture the diverse unique needs of all migrant women.

#### Unclear protection measures of women involved in precarious employment

Our findings also revealed the policy gaps that exist in protecting migrant women in the workforce. There are laws targeting migrant workers at both provincial and federal level, but there is lack of well-defined protection measures for migrant women in precarious work who are exposed to, and at risk of GBV. Given that precarious employment is largely gendered and impacted by immigration status and race [32], the absence of well-defined protection measures put migrant women in precarious positions and exposes them to abuse. Considering that precarious work is mostly dominated by migrant women who are often multiply disadvantaged, the risk of under-reporting GBV is high due to fear of losing their jobs and/or residency status. Although by-standers are important in disclosure of GBV against migrant women in workplaces, their role in reporting GBV in workplaces is not clarified in the policies reviewed, and ultimately breeds reluctance in disclosing cases of abuse in workplaces for fear of repercussions for the bystander’s own residency status and employment [33].

#### Dependent migration status and GBV

The restrictions that are attached to dependent migration status amplify the powerlessness of migrant women experiencing GBV and constitute a policy gap in Canada. Migrant women often face barriers in accessing available support and remedies [21] and for women who are financially dependent on an abusive sponsor (spouse or employer), it becomes a complex problem. Although women fleeing from violent intimate relationships and abusive employers are accommodated in the policy provision, some provisions do not adequately address their needs. Inadequacies in the immigration laws and policies have been repeatedly highlighted by researchers including lengthy delays in processing applications for vulnerable women (e.g., under the Humanitarian and Compassionate class) [6, 34] and eligibility to claim social assistance and subsidized housing creates challenges for migrant women in acquiring permanent residency as they are deemed ineligible due to their reliance on social assistance. These inadequacies in the GBV-related policies and the minimal prioritization of migrant women with dependency status in obtaining secure residency status, reflect wide policy gaps with adverse effects on the health and wellbeing of migrant women.

#### GBV service provision specific to migrant women

Our findings revealed the efforts done at provincial and federal levels to ensure that women exposed to and at risk of GBV have access to support services (e.g., housing, health services, and psychosocial support services). While access to care and support is crucial in the health and wellbeing of migrant women exposed to and at risk of GBV, there are requirements to access these services that are prohibitive for migrant women who are in Canada under uncertain or precarious circumstances. For example, to access healthcare one must apply for OHIP in Ontario or BC MSP as a legal immigrant or use medical insurance which might not be accessible to newly landed immigrants. The waiting periods and documentation required which might not be accessible to newly migrant women experiencing GBV present key policy gaps.

#### Unclear identification and referral process of trafficking victims

Regardless of the federal and provincial government’s efforts to criminalize trafficking in persons (TIP) through the National Strategy to Combat Human Trafficking (National Strategy, the Criminal Code, and Immigration Refugee Protection Act (IRPA), we identified inadequacies in the identification and support for victims particularly at the provincial levels. Remarkably, despite recognition of TIP as a violation of the rights of women, there is no explicit indication of the nexus between TIP and GBV.

What we observe mostly at provincial level is the separation of trafficking and gender-based violence prevention strategies into distinct silos, with less emphasis on recognizing how TIP is also a form of GBV often experienced by migrant women. While the Ontario Anti-Human Trafficking Strategy 2020–2025 outlines the province’s efforts in combating trafficking, there is no clear mention of the deleterious effects on migrant women who are highly vulnerable, and this lack of clarity subtly equates the experiences of migrant victims of TIP to non-migrant victims. As argues by researchers [35], trafficking flourishes not because of lack of preventive mechanisms but because of the way that the policy landscape responds to it which may not be effective. In other words, the interconnectedness of trafficking and GBV against migrant women should be recognized and preventive mechanisms tailored according to their needs.

#### Unclear GBV intervention frameworks in the wake of pandemics

Evident in the policies we reviewed is the absence of an emergency or pandemic preparedness framework, either in the form, of pre-emptive policy intervention, initiative, or guidance, to anticipate or quickly respond and prevent GBV during an emergency (health or other). The on-going COVID-19 pandemic left women vulnerable to repeated victimization resulting in injuries or death [17]. Although the federal and provincial government reacted to the greater threat of GBV during the COVID-19 pandemic, there is no clear and specific framework on addressing future pandemic and emergencies that may lead to an escalation of GBV cases. While lessons can be drawn from the COVID-19 intervention strategies that were drafted, replication of interventions can be ineffective based on the type and magnitude of the pandemic and the population groups affected. Consequently, there is need for clear intervention frameworks to cater for migrant survivors of GBV during pandemics taking into consideration that each pandemic brings its own challenges.

#### Gaps in addressing cyber violence at provincial levels

While the federal government has provided a policy provision to address the effects of cyber violence through the Protecting Canadians from Online Crime Act, gaps still exist at the Provincial level. In ON and BC, inadequate attention is given to cyber violence in GBV-related policies. Based on our review, there are no specific laws or regulations that specifically speak to cyber violence against women, let alone racialized women and/or migrant women; yet cyber-violence contributes immensely to the high rate of violence against women. Policies on cyberviolence against migrant women should not be equated with other issues that are related to cyber violence such as terrorism rather they should solely focus on cyberviolence as a form of GBV. Given the close link between cyber-violence, trafficking and migration, lack of inclusion of cyberviolence in the laws and policies does not only inhibit women’s ability to seek protection from law enforcement but exacerbates migrant women’s risk to GBV (including TIP). It is important that more focus be given to cyber violence as in most cases it is used to silence victims of GBV and ultimately deter them from disclosing the abuse [36].

### Key lessons learnt

The federal government provides a ‘blueprint’ for the prevention of GBV against migrant women. Most policies at the federal level take into consideration the needs of minority groups including migrant women (often referred to as newcomers in policies). We identified two different levels of visibility in the federal policies: 1.) the naming of inequalities and, 2) the inclusion of migrant women’s voices and insights) of visibility of migrant women in GBV-related policies in Canada at both federal and provincial levels. The inclusion of intersectionality and recognition of multiple inequalities in policies is crucial and the intersecting inequalities are predominantly expressed in policies targeting the victims of GBV both in the private and public realms. While the federal government has jurisdiction over criminal law, provincial governments have responsibility for administering justice, health, and social services to GBV survivors. ON and BC have made strides in recognizing the unique needs of migrant women exposed and at risk of GBV. Although the selected provincial government has not adopted specific legislation on GBV, BC provides GBV protection under Family Law Act, and is working towards addressing the needs of migrant women in precarious work and prioritizes training and screening of domestic violence, something that the federal Divorce Act does not have [34]. Regardless of these strides, we argue that more work should be done to ensure that migrant women are supported, their unique lived experiences taken into consideration in policy development and implementation -through the enactment of a GBV-specific federal legislation.

## Discussion

The findings from this study confirm other studies’ evidence on the importance of prioritizing the needs of migrant women at risk and exposed to GBV [8, 21, 26]. This study sought to broaden our understanding of how different elements of intersectionality have been incorporated into the violence-related policies in Canada and highlight the extent to which GBV against migrant women in Canada is rooted in multiple systems of oppression and inequalities [37]. Using the IFPA framework, we have managed to reveal not only the extent to which the policies in Canada reflect the needs of migrant women but also how these policies exacerbate their vulnerabilities and insecurities, ultimately heightening their risk of GBV.

An understanding of what constitutes GBV is crucial in policy development and implementation. Based on our findings, domestic violence, family violence and sexual assault are the commonly used terms to refer to GBV. The use of these terms shows a demarcation of the public and private perpetrated violence thereby obscuring the interactions between these realms. Ultimately, this downplays the gravity of other forms of violence (e.g., trafficking, cyber-violence) that pose adverse health risks to migrant women.

The development of GBV-related federal policies that acknowledge the multiple intersecting identities and seek to support women exposed and at risk of GBV is considered a positive move and an example of a success in moving GBV and its various forms (e.g., IPV) from the private to the public realm in the public policies reviewed. However, the unique lived experiences of migrant women exposed to and at risk of GBV are not fully recognized in the policies. Consistent with other studies conducted [8, 21, 34], we argue that the failure to capture the unique experiences of migrant women exposed to and at risk of GBV by GBV-related policies may undermine women’s capacity to find satisfying solutions. Policies lacking specificity in terms of how they seek to address the needs of migrant women who in actual sense have unique lived experiences of GBV based on their immigration status can be conceptualized as both enabling GBV perpetration and constraining prevention efforts [38, 39]. Ultimately, the scant attention given to migrant women’s lived experiences of GBV, especially in the wake of the COVID-19 pandemic, ultimately exacerbates their risk of GBV, and the adverse health outcomes associated with it.

The current domestic violence leave entitlements are not inclusive of the needs of migrant survivors in precarious employment. Migrant women with precarious immigration status and in precarious employment are likely to accept deplorable working conditions and often reluctant to disclose GBV for fear of loss of job and residency status [31, 40]. As migrant women in precarious jobs lack a voice through unions or organizations and have minimal access to benefits, their access to care and support when exposed to GBV is questionable. The health impact of the nature of precarious work and the hazardous work conditions, exacerbated by the COVID-19 pandemic, and GBV compromises their wellbeing and requires urgent intervention tailor made to their needs- a component that is inadequately addressed in the policy provisions. Due to the precarity of their employment status, migrant women are caught in a double-bind–either stay in an abusive workplace environment or face further threats, abuse, and loss of income if they attempt to leave. Notably, the absence of clear channels in sanctioning perpetrators of violence in workplaces disempowers migrant women exposed to GBV and reluctance in disclosure of violence shrouds GBV in workplaces in the culture of silence- a chain that keeps migrant women under the bondage of abuse.

Although equality is recognized the GBV policies reviewed, we cannot rule out the possibility of disparate impact considering the extent to which the policies increase migrant women’s risk of abuse and exploitation [4, 41] by failing to recognize the unique needs of each group of migrant women. In most contexts, equality requires a focus on the equal opportunities for women because of systemic and historical inequalities that exist. While this is of importance, we argue that equality in GBV policies should also focus on addressing inequalities that place multiply minoritized migrant women at higher risk as compared to women discriminated in one dimension. Failure to address this may signify a problem within the implementation of these policies and the health impact on migrant women exposed and at risk of GBV [6].

Although there is a progressive shift towards enhancing the visibility of migrant women in policies, we argue that the current policy landscape obscures the voices of migrant women by using a ‘one size fits all approach’ to categorize the needs of migrant women exposed to and at risk of GBV under racialized populations. This undermines the in-group differences that exist, the unique experiences of GBV and the adverse health outcomes. Further, our findings reveal the importance of acknowledging the heterogeneity of migrant women and the importance of including their unique lived experiences in discussions that result in policy making and implementation. The invisibility of the unique lived experience of migrant survivors of GBV undermines the importance of prevention and intervention efforts.

While a gender neutrality stance in GBV policies signifies a progressive shift in preventing and responding to GBV, we argue that framing of gender-neutral policies can hide and undermine the reality of GBV against migrant women -ultimately increasing their risk of abuse. As argued by previous researchers [42], some policies may appear gender neutral on the surface but can have disproportionate effects on migrant women due to gender. The denunciation of racial profiling is an example of the disproportionate effects of gender neutrality on migrant women. This denunciation of racial and gender profiling in criminal profiling can impede efforts to curb GBV amongst the racialized populations by hiding the gender of perpetrators which is essential in developing targeted awareness campaigns and perpetrator -focused interventions. Similar to previous researchers [43], we argue that a gender-neutrality frame should be informed by context to ensure that it advances the goal of supporting prevention efforts and challenging the blanketing of survivors in one category. It goes without saying, that a gender-neutral stance alone in policies will not eliminate GBV against migrant women, instead focus should also be on challenging the biases and misrepresentation of the needs of migrant women exposed to and at risk of GBV.

## Policy recommendations

Several policy recommendations can be gleaned from the federal and provincial levels. The policy recommendations presented here are based on the findings and policy gaps described in the preceding sections (see Table 2).

**Table 2:** Recommendations.

Level	Recommendations
**Federal**	*Enact a national legal framework on GBV*: Federal government should enact GBV-specific legal framework based on recommendations targeted at responding to the unique needs of GBV survivors including migrant women.
	*Reduce barriers to access*- The Federal government should put in place strategies that promote equitable access to care and support. Frameworks guiding provision of care at provincial levels should reduce barriers that might hinder qualifying migrant survivors of GBV from accessing immediate care and support due to issues related to waiting lists and availability of supporting documentation (e.g., proof or residence) needed to apply for certain services.
	*Prioritize trafficking in GBV-related policies*. Policies should reflect the interconnectedness of trafficking and GBV and not treat them as independent health problems. The voices of trafficking victims should also be reflected in GBV-related policies as this is essential in creating pathways for the development of intervention strategies.
	*Prioritize cyberviolence*: Federal government should establish a consistent approach across all jurisdictions in criminalizing cyber violence and guide and support the drafting of provincial legislations prohibiting on-consensual distribution of intimate images- a tool that can be used by perpetrators to trap migrant women in abusive relationships.
	*Strengthen labour laws to protect migrant women in precarious employment*: Federal government should also ensure that labour laws capture the full spectrum of GBV in workplaces and cover the needs of all migrant workers, including migrant women, in precarious employment. This should be consistent across all jurisdictions.
**Provincial**	*Reduce waiting period*: The requirements of the BC MSP and OHIP should be relaxed for eligible migrant women (particularly newly landed migrants) to reduce long waiting lists and ensure that migrant survivors get immediate intervention. Reducing the waiting period would increase access to care and support with potential health benefits.
	*Re-examine childcare policies as facilitators to access*: Although ON and BC has adopted the Toward $10-a-Day: Early Learning and Child Care initiative to facilitate access for abused migrant mothers requiring care and support, there is a need to develop strategies of ensuring that long waiting lists in childcare centers are reduced so that migrant survivors can also get access to childcare. Childcare duties can deter migrant women from seeking care and support hence the need to prioritize this.
	*Utilize a multi-axis approach in policy formulation*: There is a need to prioritize the unique lived experiences of migrant women in GBV-related policies so that interventions resulting from the policy initiatives are tailored to the needs of migrant women exposed and at risk of GBV.
	*Acknowledge migrant women in precarious employment*: Although there is evidence of support for foreign employees in Ontario and BC, there is a need for the recognition of migrant women in precarious work, their vulnerability to GBV and their unique lived experience considering that they constitute the larger number of women in precarious employment.
	*Prioritize cyber-violence*: Prioritize cyber violence and trafficking given the high vulnerability of migrant women to these two forms of GBV.

## Limitations of the review

The use of an intersectional policy framework in this review helped to better understand the extent to which GBV related policies acknowledge the migrant women’s unique lived experiences of GBV. This study also has limitations. We acknowledge that we were only able to review policy documents that were publicly available, hence policy documents that were not publicly available were not included in the analysis phase. It is possible that we might have left out relevant policy documents that address GBV against migrant women in our analysis. However, transparency is recognized as critical for effective policy design and implementation so a focus on public documents is well justified. We also would like to argue that federal and provincial policy documents should be made easily accessible by the public as they are crucial not only for the implementation of the policies but also in the research studies that seek to evaluate the effectiveness of these policies in responding to the needs of migrant women.

## Conclusion

Through the IFPA and HPT framework, this article presented a policy analysis of how the needs of migrant women are reflected in the current GBV-related policies in Canada. We argued that in as much as the federal and the selected provincial policies have made effort in recognizing the needs of migrant women, gaps still exist. We identified the following gaps within the current policy landscape; in the categorization of migrant women in the policies, the neglect of migrant women in precarious work, the risk posed by dependent migration status, unclear referral pathways and identification of trafficking victims and unclear pathways of addressing cyberviolence. These gaps pose challenges not only for migrant women exposed to GBV but to practitioners as well. On reflecting from the findings reported and the gaps identified, we propose further studies be conducted with migrant women as this is extremely useful to better understand how the policies impact their varied lived experiences. Additionally, we recommend further research involving policy actors engaged in the development and implementation of policies. This will provide insights into their perspectives on the effectiveness of the current GBV policy landscape in Canada, and the identification of existing policy gaps.

## Supporting information

S1 Table Reviewed policy documents. (DOCX)
