# Supplementary material for: A policy review on the visibility of migrant women exposed to, and at risk of gender-based violence: Considerations for inclusive and equitable policies and programs in Canada
Source: PLOS Glob Public Health. 2024 Feb 16;4(2):e0002919. doi: 10.1371/journal.pgph.0002919 (PMC10871521; doi:10.1371/journal.pgph.0002919)
Supplement: S1 Table — (DOCX) [file pgph.0002919.s001.docx]

**Reviewed Policy Documents**

| Title of policy | Year | Source/link |
| --- | --- | --- |
| Federal Policies |  |  |
| Canadian Human Rights Act | 1985 | https://laws-lois.justice.gc.ca/eng/acts/h-6/FullText.html |
| Canada Health Care Policy | 1984 | https://laws-lois.justice.gc.ca/PDF/C-6.pdf |
| Criminal Code | 1985 | https://laws-lois.justice.gc.ca/eng/acts/C-46/ |
| Immigration and Refugee Protection Act | 2002 | https://laws.justice.gc.ca/eng/acts/i-2.5/FullText.html |
| Canada Labour Code | 1985 | Canada Labour Code (justice.gc.ca) |
| Protecting Canadians from Online Crime Act |  | https://laws-lois.justice.gc.ca/eng/annualstatutes/2014_31/ |
| Divorce Act | 1985 | Divorce Act (justice.gc.ca) |
| Bill S7 Zero Tolerance for Barbaric Cultural Practices Act | 2015 | Government Bill (Senate) S-7 (41-2) - Royal Assent - Zero Tolerance for Barbaric Cultural Practices Act - Parliament of Canada |
| It’s Time: Canada’s Strategy to Prevent and Address Gender Based Violence | 2017 | It's time: Canada's strategy to prevent and address gender-based violence.: SW21-172/2017E-PDF - Government of Canada Publications - Canada.ca |
| A Place to Call Home: National Housing Strategy |  | Canada's National Housing Strategy: a place to call home.: Em12-54/2018E-PDF - Government of Canada Publications - Canada.ca https://www.placetocallhome.ca/ |
| Employment Equity Act | 1995 | https://laws-lois.justice.gc.ca/eng/acts/e-5.401/FullText.html |
| Workplace Harassment and Violence Prevention Regulations | 2020 | https://laws-lois.justice.gc.ca/eng/regulations/SOR-2020-130/FullText.html |
| Poverty Reduction Act | 2019 | https://www.laws-lois.justice.gc.ca/eng/acts/P-16.81/ |
| Opportunity for All: Canada's Poverty Reduction Strategy | 2018 | https://www.canada.ca/en/employment-social-development/programs/poverty-reduction/reports/strategy.html |
| Ontario Policies |  |  |
| Ontario Immigration Act | 2015 | https://www.ontario.ca/laws/statute/15o08?search=immigration |
| Ontario Works Act | 1997 | https://www.ontario.ca/laws/statute/97o25a?search=immigration |
| Poverty Reduction Act | 2009 | https://www.ontario.ca/laws/statute/09p10?search=immigration |
| Occupational Health and Safety Amendment Act (Violence and Harassment in Workplace) | 2009 | Occupational Health and Safety Amendment Act (Violence and Harassment in the Workplace), 2009, S.O. 2009, c. 23 - Bill 168 (ontario.ca) |
| Employment Standards Act | 2000 | Employment Standards Act, 2000, S.O. 2000, c. 41 (ontario.ca) |
| Employment Protection for foreign National Act | 2009 | https://www.ontario.ca/laws/statute/09e32?search=immigration |
| Housing Services Act (Regulation 367/11- https://www.ontario.ca/laws/regulation/110367 | 2011 | Housing Services Act, 2011, S.O. 2011, c. 6, Sched. 1 (ontario.ca) |
| Residential Tenancies Act | 2006 | https://www.ontario.ca/laws/statute/06r17?search=violence+ |
| Victims' Bill of Rights | 1995 | https://www.ontario.ca/laws/statute/95v06?search=sexual |
| Anti-Human Trafficking Strategy Act | 2021 | https://www.ontario.ca/laws/statute/21a21b?search=sexual |
| Health Insurance Act (Ontario Health Insurance Plan (OHIP) | 1990 | https://www.ontario.ca/laws/statute/90h06?search=HEALTH+INSURance |
| British Columbia Policies |  |  |
| BC Housing - Women's Transition Housing and Support Program |  | https://www.bchousing.org/housing-assistance/women-fleeing-violence/womens-transition-housing-supports |
| Family Law Act | 2011 | https://www.bclaws.gov.bc.ca/civix/document/id/complete/statreg/00_11025_00_multi#section3 |
| Priority placement program |  | https://www.bchousing.org/housing-assistance/women-fleeing-violence/priority-placement-program |
| Victim Link BC |  | https://www2.gov.bc.ca/gov/content/justice/criminal-justice/victims-of-crime/victimlinkbc |
| Crime Victim Assistance Programme |  | https://www2.gov.bc.ca/gov/content/justice/criminal-justice/bcs-criminal-justice-system/if-you-are-a-victim-of-a-crime/victim-of-crime/financial-assistance-benefits |
| Victim Services and Violence Against women Program Directory |  | https://www2.gov.bc.ca/gov/content/justice/criminal-justice/bcs-criminal-justice-system/if-you-are-a-victim-of-a-crime/victim-of-crime/victim-services-directory |
| Permanent Paid Sick Leave |  | https://www2.gov.bc.ca/gov/content/employment-business/employment-standards-advice/paid-sick-leave#eligibility |
| Domestic and Sexual Violence Leave |  | https://news.gov.bc.ca/releases/2020LBR0011-000363 |
| TogetherBC: British Columbia's Poverty Reduction Strategy | 2019 | https://www2.gov.bc.ca/gov/content/governments/about-the-bc-government/poverty-reduction-strategy |
| Child Care Benefit |  | Affordable Child Care Benefit - Province of British Columbia (gov.bc.ca) |
| The Office to Combat Trafficking in Persons (OCTIP)----Office to Combat Trafficking in Persons Toolkit Guide |  | https://www2.gov.bc.ca/gov/content/justice/criminal-justice/victims-of-crime/human-trafficking/publications-resources |
| Sexual Violence and Misconduct Policy Act | 2016 | https://www2.gov.bc.ca/assets/gov/education/post-secondary-education/institution-resources-administration/5233_sexual_violence_and_misconduct_policy_guidelines_web.pdf |
